# Modeling the Contributions of Ca^2+^ Flows to Spontaneous Ca^2+^ Oscillations and Cortical Spreading Depression-Triggered Ca^2+^ Waves in Astrocyte Networks

**DOI:** 10.1371/journal.pone.0048534

**Published:** 2012-10-31

**Authors:** Bing Li, Shangbin Chen, Shaoqun Zeng, Qingming Luo, Pengcheng Li

**Affiliations:** 1 Britton Chance Center of Biomedical Photonics, Wuhan National Laboratory for Optoelectronics-Huazhong University of Science and Technology, Wuhan, People’s Republic of China; 2 Key Laboratory of Biomedical Photonics of Ministry of Education, Department of Biomedical Engineering, Huazhong University of Science and Technology, Wuhan, People’s Republic of China; IRB Barcelona, Parc Cientific de Barcelona and CIBERNED (ISCIII), University of Barcelona, Spain

## Abstract

Astrocytes participate in brain functions through Ca^2+^ signals, including Ca^2+^ waves and Ca^2+^ oscillations. Currently the mechanisms of Ca^2+^ signals in astrocytes are not fully clear. Here, we present a computational model to specify the relative contributions of different Ca^2+^ flows between the extracellular space, the cytoplasm and the endoplasmic reticulum of astrocytes to the generation of spontaneous Ca^2+^ oscillations (CASs) and cortical spreading depression (CSD)-triggered Ca^2+^ waves (CSDCWs) in a one-dimensional astrocyte network. This model shows that CASs depend primarily on Ca^2+^ released from internal stores of astrocytes, and CSDCWs depend mainly on voltage-gated Ca^2+^ influx. It predicts that voltage-gated Ca^2+^ influx is able to generate Ca^2+^ waves during the process of CSD even after depleting internal Ca^2+^ stores. Furthermore, the model investigates the interactions between CASs and CSDCWs and shows that the pass of CSDCWs suppresses CASs, whereas CASs do not prevent the generation of CSDCWs. This work quantitatively analyzes the generation of astrocytic Ca^2+^ signals and indicates different mechanisms underlying CSDCWs and non-CSDCWs. Research on the different types of Ca^2+^ signals might help to understand the ways by which astrocytes participate in information processing in brain functions.

## Introduction

For the past few decades, the role of astrocytes has been thought to be restricted to passive, histological support elements in the central nervous system [1]. However, new functions of astrocytes have recently been identified [2], [3]. Astrocytes can release chemical transmitters that regulate synaptic transmission, activate neurons, and influence cerebral microcirculation [4], [5], and their dysfunction is implicated with neurological conditions such as epilepsy and Alzheimer’s disease [6], [7]. Ca^2+^-mediated signals are the predominant model of communication between astrocytes [8]. Two main types of Ca^2+^ responses are observed in astrocytes, including Ca^2+^ oscillations and Ca^2+^ waves [9]. Ca^2+^ oscillations are characterized as transient Ca^2+^ increases that are restricted to the single cells [10], whereas Ca^2+^ waves are characterized as Ca^2+^ elevations propagating within and between neighboring astrocytes [11]. Ca^2+^ waves in the astrocyte networks are considered to represent an effective form of intercellular signaling in the central nervous system [12].

Many experiments have suggested that Ca^2+^ oscillations in astrocytes are based on inositol 1,4,5-trisphosphate (IP_3_) receptor/Ca^2+^ channels (IP_3_R) [13], [14]. The opening of these channels can release Ca^2+^ from internal stores of the endoplasmic reticulum (ER), in a process known as calcium-induced calcium release (CICR). In addition to the Ca^2+^ released from internal stores, Ca^2+^ influx from the extracellular fluid is also reported to be needed to generate Ca^2+^ oscillations [15] and voltage-gated calcium channels (VGCCs) have been found to contribute to this Ca^2+^ influx [13], [16]. However, other works show that extracellular Ca^2+^ is not required for the occurrence of Ca^2+^ oscillations [17]. Ca^2+^ released from the ER is usually considered to be the key factor in the generation of Ca^2+^ waves which are induced by ATP or IP_3_ [18], [19], but this is not necessary when Ca^2+^ waves are triggered by cortical spreading depression (CSD) [20], [21]. CSD refers to a pathophysiological phenomenon and manifests as a self-propagation wave of electrical silence, resulting in the depolarization of neurons and astrocytes and a redistribution of ions [22], [23], which is thought to underlie the migraine aura and develop after cerebral ischemia and trauma [24], [25]. The contradictive results about astrocytic Ca^2+^ signals suggest that the underlying mechanisms in the generation of Ca^2+^ oscillations and Ca^2+^ waves are still unclear.

Interestingly, spontaneous Ca^2+^ oscillations (CASs) and CSD-triggered Ca^2+^ waves (CSDCWs) have been reported in the same experiments [21]. Different models have been used to investigate the mechanisms of astrocytic Ca^2+^ signals. However, these models either focused just on CASs [26], [27], or on Ca^2+^ waves induced by ATP or IP_3_ but not by CSD (non-CSDCWs) [28]–[30]. Bennett et al. simulated the CSDCWs, but Ca^2+^ flows within the astrocytes, for example, the Ca^2+^ released from CICR and the Ca^2+^ uptaken into the ER, were neglected in their model [31]. In addition, the role of Ca^2+^ from the ER for the generation of different types of Ca^2+^ waves (CSDCWs and non-CSDCWs) could not be fully explained by these models.

In the present study, we investigated CASs and CSDCWs in a one-dimensional astrocyte network by an expanded version of our previous model, which simulated the VGCCs-mediated CASs [32], to account for the contributions of different Ca^2+^ flows between the extracellular space, the cytoplasm and the ER of astrocytes to the generation of these Ca^2+^ signals. We first explored the mechanisms for the generation of CASs and CSDCWs, and then investigated the interactions between CASs and CSDCWs, and finally addressed the transition from CASs to CSDCWs. Our results quantitatively analyze the generation of astrocytic Ca^2+^ signals and indicate different mechanisms underlying CSDCWs and non-CSDCWs.

## Methods

**Figure 1:**
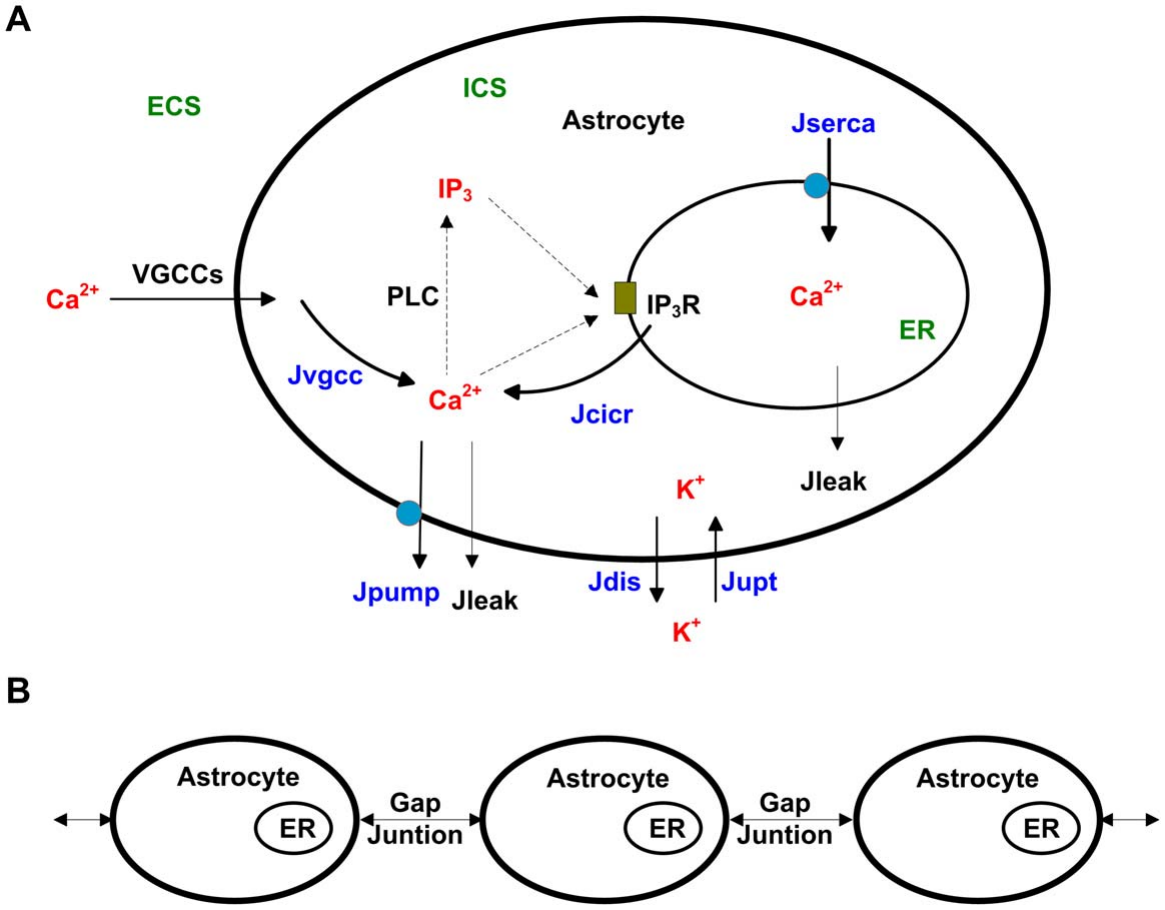
A schematic diagram of the model. (A) As to the single astrocyte, Ca^2+^ influx through voltage-gated calcium channels (VGCCs) triggers the fluctuation of Ca^2+^ in the intracellular space (ICS), enhancing the production of inositol 1,4,5-triphosphate (IP_3_), which is catalyzed by phospholipase C (PLC). Ca^2+^ and IP_3_ bind to IP_3_ receptors (IP_3_R), activating the process of calcium-induced calcium release (CICR). The endoplasmic reticulum (ER) is filled with Ca^2+^ by the sarcoplasmic/endoplasmic reticulum calcium ATPase (SERCA). A Ca^2+^ pump discharges Ca^2+^ from the ICS into the extracellular space (ECS). K^+^ in the ECS is partly uptaken into the ICS during cortical spreading depression (CSD). J_VGCC_, J_CICR_ and J_SERCA_ represent the Ca^2+^ flow through VGCCs, CICR and SERCA, respectively. J_pump_ represents the Ca^2+^ flow through the Ca^2+^ pump. J_leak_ represents the leak Ca^2+^ flow. J_upt_ represents the K^+^ flow untaken into ICS, and J_dis_ represents the K^+^ flow discharged into ECS. (B) Single astrocytes are coupled to the adjacent ones by the transfer of IP_3_ from cytosol to cytosol through gap junctions to form a one-dimensional astrocyte network.

The model consisted of a single lane of astrocytes which were assumed as spherical somas with a radius of 5 *µ*m. In a single-cell context, three compartments were considered, including the extracellular space (ECS), the intracellular space (ICS), and the ER internal space, as seen in Fig. 1A. As to the astrocyte networks, astrocytes were coupled to the adjacent ones by the transfer of IP_3_ from cytosol to cytosol through gap junctions (Fig. 1B).

### Ca^2+^ Flows through the Astrocytic Membrane

VGCCs include high-voltage-activated channels and low-voltage-activated channels. Low-voltage-activated channels have been demonstrated to have little effect on CASs and CSDCWs [16], [31]–[33]. In the present model, only high-voltage-activated channels (as a group) were considered. Similar operations were also applied by other groups [31], [33] in the study of CSDCWs. The Hodgkin-Huxley equation was used to model VGCCs:
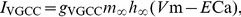
(1)where, *I*_VGCC_ is the Ca^2+^ current that flows into astrocytes via VGCCs, and *g*_VGCC_ is the membrane conductance. As shown in [31], *m*_∞_ and *h*_∞_ are gated parameters that regulate the activation and inactivation of the VGCCs, respectively.
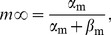
(2a)where



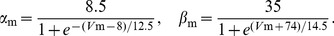
(2b)

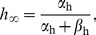
(3a)where
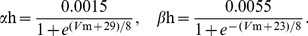
(3b)
*V*m is the astrocytic membrane potential, and its calculation is defined in the following text. ECa is the Nernst potential of Ca^2+^.
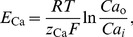
(4)where, R is the ideal gas constant, T is the absolute temperature, z_Ca_ is the valence of Ca^2+^, and F is the Faraday constant. *Ca*_o_ and *Ca*_i_ represent the Ca^2+^ concentration in the ECS and in the ICS, respectively. According to [32], the Ca^2+^ current of VGCCs described in Eq. (1) was converted into flux to calculate its contribution to the increase of Ca^2+^ in astrocytes:
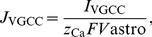
(5)where, *V*_astro_ is the volume of an astrocyte.

Ca^2+^ in astrocytes is partly discharged into the ECS by the Ca^2+^ pump, and the calculation is adapted from [31]:
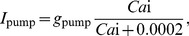
(6)where *g*_pump_ is the membrane conductance of the pump and additionally, the current in Eq. (6) was also converted into flux:



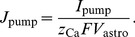
(7)The leak flux into the ECS was calculated following [26], [32]:
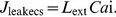
(8)where, *L*_ext_ is the rate of Ca^2+^ efflux from astrocytes.

### Ca^2+^ Flows through the Membrane of ER

As shown in Fig. 1A and in our previous model [32], Ca^2+^ flows through the membrane of ER included Ca^2+^ released from CICR, Ca^2+^ uptaken into the ER via the sarcoplasmic/endoplasmic reticulum Ca^2+^ ATPase (SERCA) and leak flux through the membrane of ER, which are described as *J*_CICR_, *J*_SERCA_ and *J*_leakics_ in Eqs. (S1)-(S3) of Materials S1, respectively. Combining Eqs. (5), (7), (8) and Eqs. (S1–S3), the dynamics of Ca^2+^ in the astrocytic cytosol were expressed as
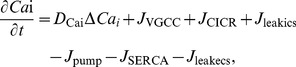
(9)where, *D*_Cai_ is the diffusion coefficient for Ca^2+^ in the cytosol and Δ is the Laplace operator. The first term on the right of Eq. (9) represents the diffusion of Ca^2+^ in the ICS. Accordingly, Ca^2+^ in the ER (*Ca*_ER_) was determined by



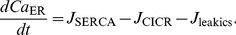
(10)The dynamics of *Ca*_o_ were neglected owing to its slight changes during Ca^2+^ oscillations in previous models. However, *Ca*_o_ changes dramatically during CSD [23], [34]. Combining Eqs. (5), (7), (8) and the influence from CSD, Ca^2+^ in the ECS was modeled by
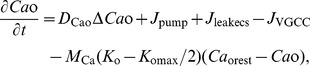
(11)where, *D*_Cao_ is the diffusion coefficient for Ca^2+^ in the ECS. *K*_o_ represents the K^+^ concentration in the ECS. *M*_ca_ is a constant, *K*_omax_ is the maximal *K*_o_ during CSD, and *Ca*_orest_ is *Ca*_o_ at resting state. The first term on the right of Eq. (11) represents the diffusion of Ca^2+^ in the ECS. The last term on the right of Eq. (11) describes the decrease of *Ca*_o_ during CSD, and its precise mechanism needs to be further explored.

### IP_3_ in the ICS

IP_3_ is a known intracellular messenger, which can bind to the IP_3_R to cause Ca^2+^ to flow out of the ER. In a single-cell context, IP_3_ in the ICS (*IP*_3_) was catalyzed by phospholipase C (PLC) as defined in Eq. (S4) of Materials S1. Experimental evidences show that Ca^2+^ waves in astrocytes are mediated following the transfer of IP_3_ [35], [36]. In the present astrocyte networks, the astrocyte was coupled to its nearest neighbors by the transfer of IP_3_ through gap junctions. Following [37], the change of *IP*_3_ in astrocyte *i* due to the gap junction with astrocyte *j* is
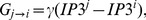
(12)where *i*, *j* are indices of adjacent astrocytes, and *γ* is the coupling strength. Combining the diffusion of IP_3_ inside the cells [38] and Eq. (S4), IP_3_ in astrocyte *i* was calculated as
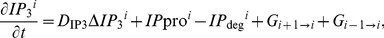
(13)where, *D_IP3_* is the diffusion coefficient for IP_3_ in the ICS. *IP_pro_* represents the production of IP_3_ and *IP*_deg_ represents its degradation, as seen in Eq. (S4) of Materials S1.

### Cortical Spreading Depression

CSD causes neurons and astrocytes to depolarize and greatly changes the ion concentration [23], [34]. We incorporated CSD in this model to initiate astrocytic Ca^2+^ waves. Because high extracellular K^+^ is required for the propagation of CSD [39], *K*_o_ was described as the classical diffusion-reaction equation:
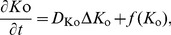
(14a)where, *D*_Ko_ is the diffusion coefficient for K^+^ in the ECS, and the first term on the right of Eq. (14a) represents the diffusion of K^+^ in the ECS. *f*(*K*_o_) describes the reaction process of *K*_o_, which is adapted from [40]:
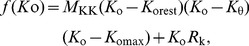
(14b)where, *M*_KK_ is a rate constant, *K*_orest_ is *K*_o_ at the resting level, and *K*_θ_ is the threshold for the triggering of CSD. The first term on the right of Eq. (14b) meets the following requirements: maintaining homeostasis at the resting level, triggering explosive subsequent growth in *K*_o_ when *K*_o_ is higher than the threshold *K*_θ_, and preventing *K*_o_ rising when *K*_o_ is beyond the ceiling Komax [40]. The second term represents the recovery of *K*_o_. *R*_k_, which restores *K*_o_ to the normal level, was modeled by:
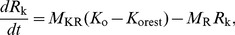
(15)where, *M*_KR_ and *M*_R_ are constants.

Astrocytes are reported to be fast buffers and play an important role in the clearance of excess *K*_o_ [41], [42]. During the process of CSD, *K*_o_ would partly be untaken by astrocytes. The K^+^ concentration in the astrocytes (*K*_i_) was calculated as.
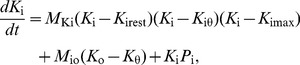
(16)where, *M*_Ki_ and *M*_io_ are rate constants. *K*_irest_ is *K*_i_ at the resting level, *K*_iθ_ is the threshold for the fast elevation of *K*_i_, and *K*_imax_ is the ceiling *K*_i_ during CSD. The first term on the right of Eq. (16) follows the formalism of *K*_o_ to maintain cytosol K^+^ homeostasis, the senond term is used to detect the change in *K*_o_ and is assumed to be effective only when *K*_o_ is beyond the threshold for the triggering of CSD, and the third term represents the discharge of *K*_i_.
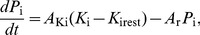
(17)where, *A*_Ki_ and *A*_r_r are constants. The first term on the right of Eq. (17) represents the discharge of *K*_i_, and the second is a decay term.

The astrocytic membrane potential is a complicated parameter to calculate in computational models. To avoid these complex calculations, a simplified method was adopted. It is well known that the K^+^ Nernst potential is close to *V*_m_; therefore, in this study, the K^+^ Nernst potential was used to approximate *V*_m_ by adding a modulation factor, which was chosen based on previously experiments [43].
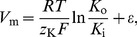
(18)where, *z*_K_ is the valence of K^+^ and *ε* is the modulation factor.

### Implementation

The chain model consisted of 3*N* astrocytes, where *N* ranged from 1 to 100. All of the computations and visualizations of this model were implemented in the Matlab environment (Matlab 7.0, MathWorks Inc., USA). The Crank-Nicholson algorithm was used to solve the differential equations [44], with a zero-flux boundary condition and a time step of 15 ms. The parameter values used in the model are shown in Table S1, and initial values of the variables are list in Table S2.

## Results

### The Contributions of Different Ca^2+^ Flows to CASs

**Figure 2:**
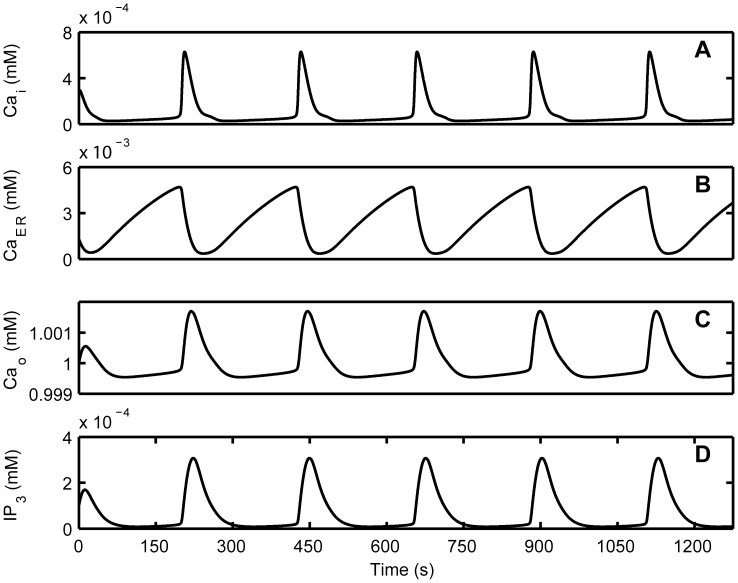
Characteristics of spontaneous Ca^2+^ oscillations (CASs) in the computerized astrocytic model. (A) Ca^2+^ oscillations in the ICS occur without any stimulus. (B) Ca^2+^ oscillations in the ER occur earlier and last longer than those in the ICS. (C) The concentration of Ca^2+^ in the ECS decreases slightly before Ca^2+^ oscillations in the ICS and then increases after the oscillations. (D) IP_3_ oscillates in the ICS.

In this model, CASs occurred in single astrocytes without any stimulus. Fig. 2A shows that in the ICS, cytosol Ca^2+^ oscillated with the amplitude of 0.63×10^−3^ mM and the frequency of 0.0044 Hz, which were consistent with the experimental results [17], [45]. The duration of cytosol Ca^2+^ oscillations, measured at the half-amplitude level, was 21 s. In the ER (Fig. 2B), the oscillations of Ca^2+^ showed a larger amplitude and a longer duration than those in the ICS. As been reported in the experiments [46], [47], the peak time point for oscillations came earlier in the ER than in the ICS (Fig. 2A, B). The duration of IP_3_ fluctuation in the ICS was longer than that of Ca^2+^ oscillations in the ICS but shorter than that of Ca^2+^ oscillations in the ER (Fig. 2D). In the ECS, a slight decline in Ca^2+^ was noticed before each Ca^2+^ oscillation in the ICS (Fig. 2C). The change of Ca^2+^ concentration in the ECS indicated that CASs in the ICS are related to the Ca^2+^ in the ECS [15], [16].

CASs in the ICS depended on Ca^2+^ influx from the ECS and Ca^2+^ released from the ER [13], [15], [16], but the precise contributions of these Ca^2+^ flows are not clear. Here, we investigated the influence of different Ca^2+^ flows on the generation of CASs. In this model, CICR that released Ca^2+^ to the ICS and SERCA that extracted Ca^2+^ to the ER were considered to be the main processes that modulated the exchange of Ca^2+^ between the ICS and the ER. VGCCs were thought to regulate extracellular Ca^2+^ that flowed into the ICS. To understand the contributions of these different processes, we inhibited CICR, SERCA, and VGCCs separately to compare with the control condition, which had no inhibition of CICR, SERCA, and VGCCs.

**Figure 3:**
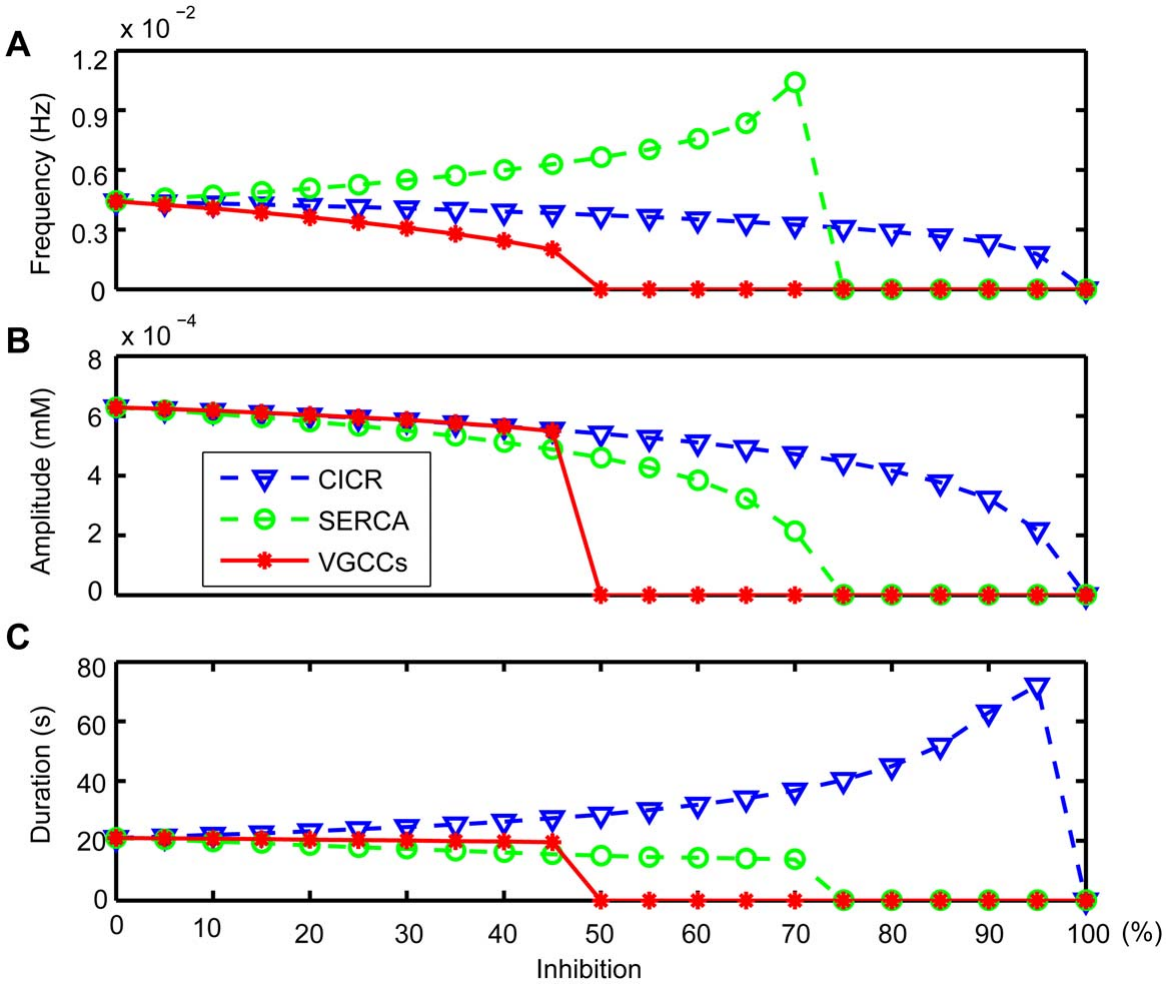
The influence of different Ca^2+^ flows on CASs. By inhibiting CICR (dashed triangle), the frequency (A) and amplitude (B) of CASs decrease, but the duration (C) increases. CASs do not occur when CICR is inhibited more than 95%. By inhibiting SERCA (dashed circle), the frequency (A) of CASs increases, but both the amplitude (B) and the duration (C) decrease. CASs do not occur when SERCA is inhibited more than 70%. Inhibiting VGCCs (solid star) has little effect on the amplitude (B) and duration (C) but great on frequency (A) before CASs disappear. CASs do not occur when VGCCs are inhibited more than 45%.

The CICR inhibition was achieved by reducing the release of Ca^2+^ from the ER (reducing *M*_CICR_ in Eq. (S1) of Materials S1). Fig. 3A shows that the frequency of CASs progressively decreased with the inhibition of CICR, consistent with the reported experimental results that CICR plays an important role in the generation of Ca^2+^ oscillations, and oscillations cannot occur without Ca^2+^ released from the ER [13], [48], [49]. In addition, CICR inhibition decreased the amplitude of CASs, whereas the duration was increased, as seen in Fig. 3B, C and in Fig. S1. The SERCA inhibition was achieved by reducing the uptake of Ca^2+^ into the ER (reducing *M*_SERCA_ in Eq. (S2) of Materials S1). SERCA inhibition decreased the ability to form large-amplitude oscillations (Fig. 3B) [13], [17], [48], as well as the duration (Fig. 3C). Previous results indicate a decline in the frequency of CASs with SERCA inhibition [16], [45], whereas our simulation results suggest that the frequency of CASs first increases (Fig. 3A) and then Ca^2+^ dynamics evolve into small oscillations before finally disappearing, as seen in Fig. S2. The membrane conductance, *g*_VGCC_, was reduced to simulate the VGCCs inhibition. After the reduction, the frequency of CASs decreased (Fig. 3A), confirming that VGCCs can mediate CASs [16], [32]. The amplitude and duration of CASs also decreased as a result of VGCCs inhibition (Fig. 3B, C). In the condition that CASs were not fully blocked, CICR or SERCA inhibition had a significant effect on the frequency, amplitude and duration of CASs, whereas VGCCs inhibition had little effect on the amplitude and duration but a great effect on the frequency, as seen in Fig. 3 and in Fig. S3. These results indicate that the elevation of Ca^2+^ in the CASs mainly came from the Ca^2+^ released from internal stores.

### The Contributions of Different Ca^2+^ Flows to CSDCWs

**Figure 4:**
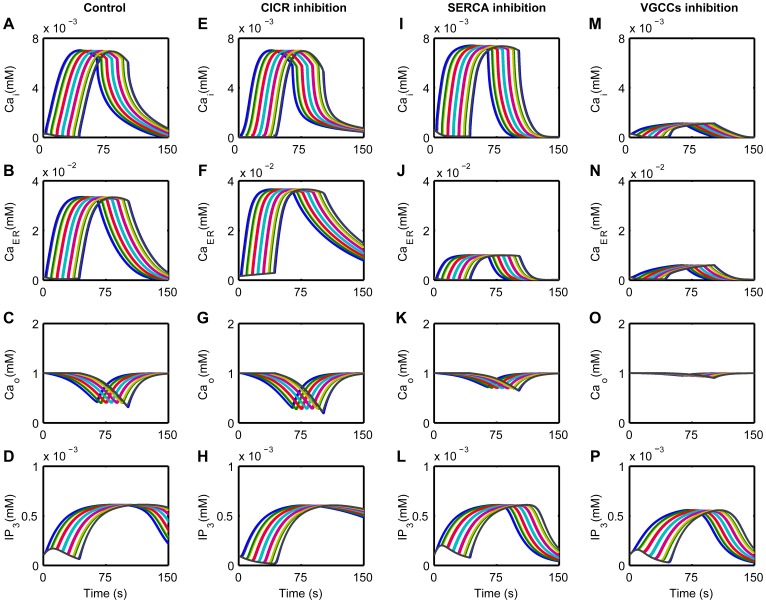
The influence of different Ca^2+^ flows on CSD-triggered Ca^2+^ waves (CSDCWs). A typical CSDCW is characterized as the significant elevation of Ca^2+^ in the ICS at successive astrocytes in the network (A), associated with the increase of Ca^2+^ in the ER (B), the increase of IP_3_ in the ICS (D) and the decrease of Ca^2+^ in the ECS (C). CICR inhibition shortens the duration of increased Ca^2+^ in the ICS (E), slows the recovery of Ca^2+^ in the ER (F), decreases Ca^2+^ in the ECS more than in the control condition (G) and increases the duration of increased IP_3_ in the ICS (H). SERCA inhibition increases the amplitude of Ca^2+^ in the ICS (I) and decreases that in the ER (J). Ca^2+^ in the ECS is increased compared to the control (K). Because high Ca^2+^ in the ICS would inhibit the process of CICR, the increase of IP_3_ in the ICS is shortened (L). After VGCCs inhibition, Ca^2+^ is largely weakened in the ICS (M), in the ER (N) and in the ECS (O). The changes of IP_3_ are also shortened because of the low Ca^2+^ in the ICS (P).

According to Kager et al. and Chapuisat et al. [50], [51], local extracellular K^+^ concentration (around the central astrocytes in the astrocyte networks in this model) was elevated to 12 mM to evoke a CSD. CSD would cause the local extracellular K^+^ to increase further and spread to the surrounding [50], [51]. Usually, the propagation of CSD is indicated as the spread of significantly increased *K*_o_ [40]. As shown in Fig. S4A, *K*_o_ was significantly increased and spread in this model after locally increasing extracellular K^+^ concentration, suggesting the emergence of CSD, and then *K*_i_ was also increased to partly clear the excess K^+^ in the ECS (Fig. S4B). Owing to the depolarization of astrocytes caused by CSD (Fig. S4C), Ca^2+^ influx via VGCCs increased. A Ca^2+^ wave was induced by CSD and propagated in the astrocyte networks at a speed of 58 *µ*m/s, with the amplitude of 6.4×10^−3^ mM and the duration of 56.7 s, as seen in Fig. 4A. The amplitude and duration of CSDCWs were significantly larger than those of CASs in the ICS.

It was readily apparent that Ca^2+^ levels in the ER and in the ECS changed as the CSDCWs spread (Fig. 4B, C). Here, we also took into account the processes of CICR, SERCA and VGCCs to study the contributions of different Ca^2+^ flows to the generation of CSDCWs.

Compared to the control condition, the CICR inhibition shortened the duration of the increased *Ca*_i_ (Fig. 4E). Due to that Ca^2+^ could not be rapidly released from the ER through the process of CICR, the rate of recovery of *Ca*_ER_ was reduced (Fig. 4F). To maintain the amplitude of Ca^2+^ in the ICS, more Ca^2+^ in the ECS was needed to flow into the ICS to make up for the reduction of Ca^2+^ released from the ER, causing *Ca*_o_ to decrease further (Fig. 4G) and the duration of *IP_3_* elevation to be increased (Fig. 4H). After SERCA inhibition, Ca^2+^ in the ICS could not be extracted into the ER promptly by SERCA, and this led to a decrease in *Ca*_ER_ (Fig. 4J) and an accumulation of *Ca*_i_ (Fig. 4I). Owing to this accumulation, Ca^2+^ influx was reduced, and *Ca*_o_ was increased compared to the control (Fig. 4K). Because high Ca^2+^ in the ICS inhibits the process of CICR [29], the duration of *IP_3_* elevation in the ICS was shortened (Fig. 4L). After VGCCs inhibition, the changes of Ca^2+^ were strongly weakened in the ICS, in the ER and in the ECS (Fig. 4M, N and O). Compared to the control (Fig. 4D), the duration of *IP_3_* elevation was also shortened owing to low Ca^2+^ in the ICS (Fig. 4P). These results suggest that CSDCWs are primarily triggered by Ca^2+^ influx via VGCCs, and the Ca^2+^ efflux from the ER contributes to the generation of Ca^2+^ waves to a lesser degree.

### Interactions between CASs and CSDCWs

**Figure 5:**
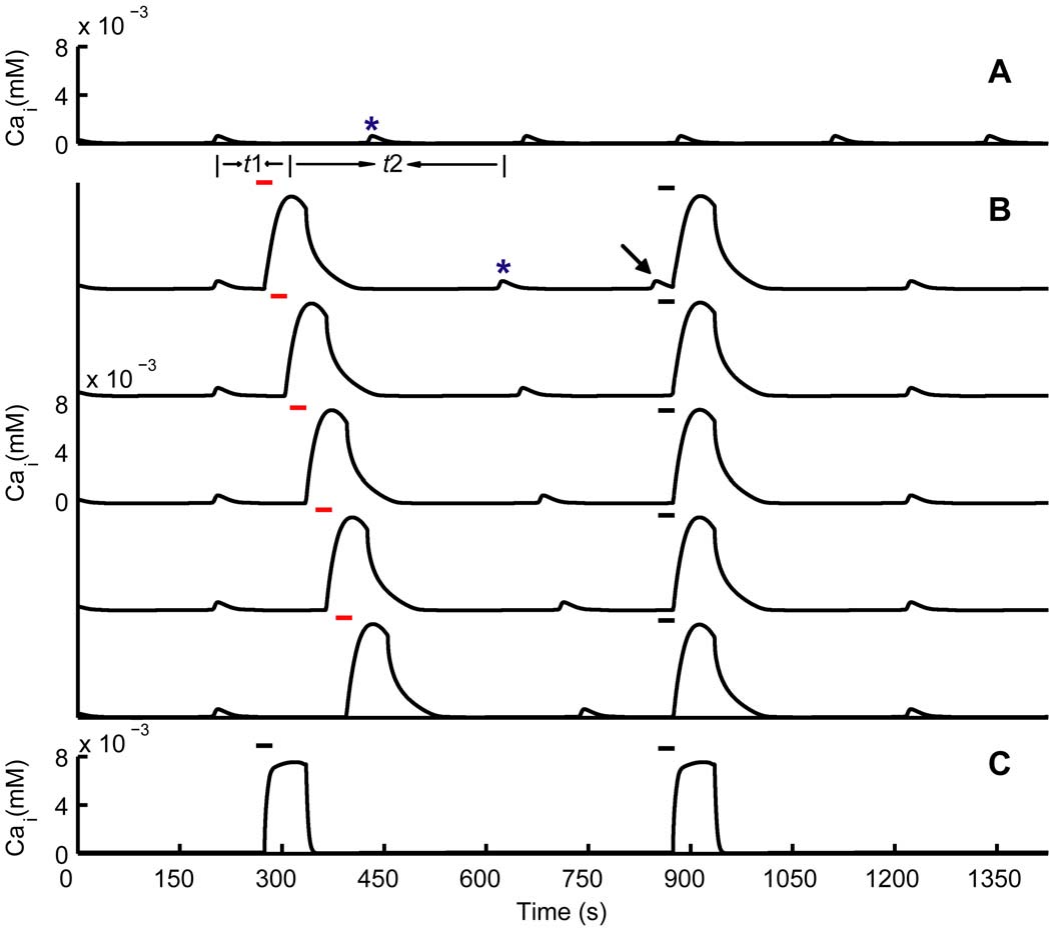
Interactions between CASs and CSDCWs. (A) A series of CASs occur without any stimulus. (B) The appearance of CSDCWs depresses CASs, and CASs reappear a few minutes after the pass of CSDCWs (the affected CASs are marked with asterisks in A and B). CSDCWs can appear immediately after CASs (marked with an arrow in B). By regulating the appearance time of CSDCWs through changing the time points of locally elevating *K*_o_ (marked with red bars in B), the peak-to-peak interval between the CSDCW and the following CAS is similar, and it is not related to the peak-to-peak interval between the CSDCW and the previous CAS. *t*1, the peak-to-peak interval between the CSDCW and the previous CAS. *t*2, the peak-to-peak interval between the CSDCW and the following CAS. (C) Depletion of Ca^2+^ stores in the ER abolishes CASs, but CSDCWs still spread. The bar illustrates the time of locally elevating *K*_o_.

Experiments showed that the appearance of CSDCWs depressed CASs, and then CASs reappeared after the pass of CSDCWs in single astrocytes [21]. To investigate the interactions between CASs and CSDCWs in this study, we focused on Ca^2+^ signals in single astrocytes, and the results were similar to other astrocytes which underwent CASs and CSDCWs in the astrocyte networks. Comparing Fig. 5A with Fig. 5B, it shows that the CAS (marked with an asterisk in Fig. 5A, B) was absent after the appearance of CSDCW, and reappeared a few minutes later after the peak of the CSDCW. Moreover, CSDCWs could be induced immediately after the occurrence of the CAS (marked with an arrow in Fig. 5B), which indicated that CASs did not prevent the generation of CSDCWs. To further understand the effect of CSDCWs on CASs, the appearance time of CSDCWs was manipulated, by changing the time points of locally elevating extracellular K^+^ concentration (marked with red bars in Fig. 5B), to investigate the changes in peak-to-peak interval between the CSDCW and the following CAS (see *t*2 in Fig. 5B). Fig. 5B shows that by regulating the appearance time of CSDCWs, the peak-to-peak interval between the CSDCW and the following CAS remained almost constant. This suggests that CSDCWs had a similar effect on the latency to the onset of the following CAS, and the latency was not affected by the appearance time of CSDCWs relative to the previous CAS (see *t*1 in Fig. 5B). By depleting the Ca^2+^ store, CASs were completely abolished, but CSDCWs still propagated with shorter duration (Fig. 5C), suggesting that there were different mechanisms underlying the generation of CASs and CSDCWs. Furthermore, CSDCWs could spread without Ca^2+^ flow from ER, which also indicates different mechanisms underlying CSDCWs and non-CSDCWs [21].

### Transition from CASs to CSDCWs

**Figure 6:**
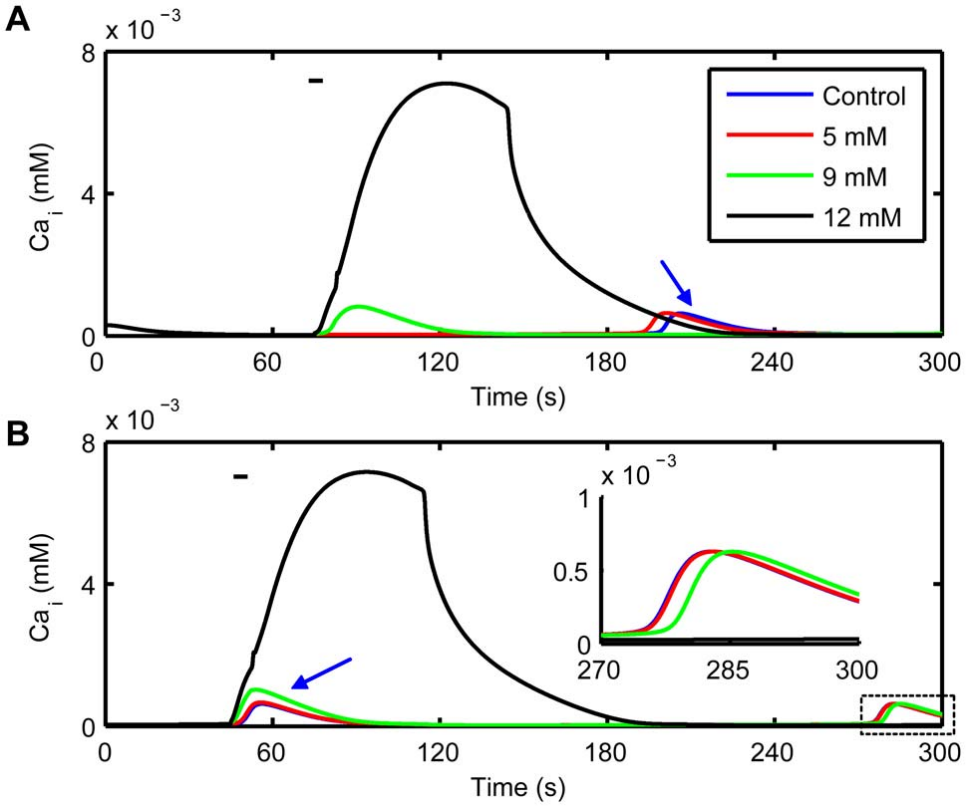
Transition from CASs to CSDCWs. (A) By locally increasing *K*_o_ to 5 or 9 mM, Ca^2+^ in ICS is increased, which facilitates the occurrence of CASs. When *K*_o_ is increased to 12 mM, a CSDCW is induced. (B) Increasing *K*_o_ in the present of CASs elevates Ca^2+^ in ICS and then the elevated Ca^2+^ will postpone the occurrence of the following CASs. Following oscillations are shown in the illustration. The bar illustrates the time of locally elevating *K*_o_, the concentrations of which are shown in the legend. Arrows indicate the appearance time of CASs under the control condition.

Local *K*_o_ (around the central astrocytes in the astrocyte networks in this model) was increased gradually to explore the transition from CASs to CSDCWs in single astrocytes. It shows that increasing *K*_o_ would induce the elevation of *Ca*_i_ and the generation of CASs in the astrocytes near to the stimulation site (Fig. 6A), and higher *K*_o_ caused an earlier onset time for oscillations compared to the control (the onset of CAS under the control condition is marked with an blue arrow). By increasing *K*_o_ in the present of CASs facilitated the further elevation of *Ca*_i_ and the elevated *Ca*_i_ deferred the occurrence of successive oscillations (Fig. 6B and its illustration). When *K*_o_ was increased to 12 mM, a CSDCW was evoked and propagated to neighboring astrocytes.

## Discussion

In this model, we incorporated CASs and CSDCWs in a one-dimensional astrocyte network, and investigated the contributions of different Ca^2+^ flows, including Ca^2+^ flows between the extracellular space, the cytoplasm and the ER of astrocytes, to the generation of these Ca^2+^ signals. The results show that CASs depended primarily on Ca^2+^ released from internal stores in astrocytes, whereas CSDCWs depended mainly on voltage-gated Ca^2+^ influx. The appearance of CSDCWs would suppress CASs, whereas CASs did not prevent the generation of CSDCWs. Furthermore, our results suggest that after Ca^2+^ stores have been depleted, CSDCWs could still propagate due to voltage-gated Ca^2+^ influx, different from the non-CSDCWs.

The predominant model of communication between astrocytes is Ca^2+^-mediated signals, which are determined by an intricate interplay between Ca^2+^ influx, buffering and extrusion pathways [8]. Experimental results show that CASs require extracellular Ca^2+^ and operating VGCCs [16], whereas others suggest that single CASs are observed many minutes later after the elimination of extracellular Ca^2+^ [17]. Our simulations support the former and furthermore, we show that in the present of CASs, both CICR inhibition and SERCA inhibition had a significant effect on the amplitude and duration of CASs, whereas VGCCs inhibition had little effect on the amplitude and duration but a great effect on the frequency. It indicated that the elevation of Ca^2+^ in the CASs mainly came from the Ca^2+^ released from the ER, and that Ca^2+^ influx from the ECS might play a role in triggering the process of CICR to generate CASs and replenishing the Ca^2+^ load during CASs [27].

In situ and in vivo experiments show that CASs occur 0.15 to 1 time per minute (0.0025 to 0.017 Hz) [17], [52]–[54]. The frequency of CASs in our model is 0.0044 Hz, which is within the reported frequency range and suggests that the modeled CASs here recapitulate the physiological astrocytic Ca^2+^ responses. Local Ca^2+^ transients in the astrocytic processes have mean frequency of 0.028 Hz, which is significantly higher than the frequency of Ca^2+^ transients in the astrocyte cell body [55]. Although Ca^2+^ transients in the processes of astrocytes are not considered in the present model, the generation of Ca^2+^ transients in the processes of astrocytes is reported to depend mainly on the Ca^2+^ released from internal stores [55], which is similar as the CASs in the astrocyte cell body in our model.

Ca^2+^ waves in astrocytes are usually thought to be induced by ATP or IP_3_, and they depend on Ca^2+^ released from internal stores [18], [19]. This kind of Ca^2+^ waves was represented as non-CSDCWs in this study. There are at least three differences between the CSDCWs and non-CSDCWs. First, after depletion of internal stores, non-CSDCWs cannot be generated [18], [19], while CSDCWs could still propagate [21]. Second, theoretical and experimental results suggest that CSDCWs have a larger amplitude than that of non-CSDCWs [21], [28], [31]. Third, CSDCWs spread faster than non-CSDCWs [11], [21]. All of these suggest that there may be different mechanisms underlying CSDCWs and non-CSDCWs. In this model, the amplitude and speed of CSDCWs were similar to reported results [31], significantly larger than those of non-CSDCWs [11], [28]. Moreover, by depleting the internal store in this study, CSDCWs could still propagate. This is because that even without Ca^2+^ efflux from the internal stores, CSD can cause astrocytes to depolarize, which opens the VGCCs, and Ca^2+^ influx via VGCCs is able to generate Ca^2+^ waves. It suggests that the generation of CSDCWs depends primarily on Ca^2+^ influx via VGCCs. VGCCs have been reported to be not physiologically relevant for intracellular Ca^2+^ signals [56], while CSDCWs pertain not to this case, because CSD would cause astrocytes to depolarize and Ca^2+^ influx coupled to depolarization was recorded during CSDCWs [21]. It is reported that after CSD has stopped, which means that astrocytes do not depolarize and no membrane current are detectable, Ca^2+^ waves still spread but with a significantly reduced amplitude and speed [21], suggesting that non-CSDCWs might depend mainly on Ca^2+^ flows uncoupled to depolarization. In this study, we only simulated CSDCWs, focusing on the voltage-gated Ca^2+^ influx pathway, and this might explain why the amplitude of CSDCWs was not strongly attenuated after depletion of internal stores compared to the experiment data [21], though the duration was reduced.

We investigated the interactions between CASs and CSDCWs, and showed that CSDCWs depressed CASs and CASs reappeared after the pass of CSDCWs, which was consistent with previous experiments [21]. Furthermore, CSDCWs postponed the onset of the following CASs with a similar time lag, and this might be related to the process of CSD. As an “all or none” process [57], CSD would elevate Ca^2+^ in the cytosol to a similar level. As a result, it took astrocytes equivalent time to recover from the effect of CSD, which might determine the latency to the onset of following CASs. Locally increasing *K*_o_ would cause astrocytes to depolarize and increase Ca^2+^ influx via VGCCs, which facilitated the occurrence of CASs and the elevation of *Ca*_i_. Meanwhile, the onset of subsequent Ca^2+^ oscillation was postponed as the elevation of *Ca*_i_. This is because that in addition to the increased amplitude of *Ca*_i_ caused by increasing *K*_o,_ the duration of *Ca*_i_ was also increased. As a result, astrocytes needed more time to recover from the elevation of *Ca*_i_, and the subsequent oscillations came later.

Ca^2+^ signals in astrocytes are quite variable in the spatiotemporal organization. CASs in cortical layer 2/3 show infrequent synchronous pattern, whereas in layer 1 CASs are frequent asynchronous [58]. CSDCWs in gray matter of the neocortex are reported to propagate with a higher speed than non-CSDCWs in white matter [21], [59]. The complexity may be related to the mechanisms that control Ca^2+^ entry from the extracellular space as well as Ca^2+^ release from internal stores. VGCCs, which are expected to play significant functional roles in Ca^2+^ influx in astrocytes [60], are key transducers of membrane potential changes into intracellular Ca^2+^ transients. According to our study, voltage-gated Ca^2+^ influx had a great contribution to CSDCWs, and might play a role in triggering CASs. However, the distribution of VGCCs expression in the brain tissue is not uniform. Experiments show apparent lack of VGCCs in rat hippocampus and visual cortex [56], while different types of VGCCs are present in mouse hippocampus [61]. Astrocytes differ in membrane currents and are heterogeneous with respect to VGCCs expression [62], but several types of astrocytes can coexist within the same brain region [63], which might lead to the heterogeneity of Ca^2+^ signals among astrocytes.

Experiments show that CASs are based on the process of CICR [13], [14], but Ca^2+^ entry via the external medium has also been found to contribute to CASs [13], [16]. In our model, we quantitatively analyzed the contributions of different Ca^2+^ flows to the generation of astrocytic Ca^2+^ signals, and showed that voltage-gated Ca^2+^ influx played a role in regulating the frequency of CASs and might be important for initiating CASs [64]. Depleting internal Ca^2+^ stores is reported to block non-CSDCWs [18], [19], but not CSDCWs [20], [21]. Consistent with the experimental results, our model showed that after Ca^2+^ stores have been depleted CSDCWs could still propagate. Furthermore, we showed that the propagation of CSDCWs after depleting internal Ca^2+^ stores was due to voltage-gated Ca^2+^ influx, indicating different mechanisms underlying CSDCWs and non-CSDCWs. Future research should focus on the different mechanisms underlying astrocytic Ca^2+^ signals.

A recent study has simulated the transition from single CASs to non-CSDCWs, and the results suggested that long-distance non-CSDCWs are favored when the internal Ca^2+^ dynamics implements the frequency modulation-encoding oscillations [28]. We explored the transition from CASs to CSDCWs and showed that CSDCWs would occur when there was enough Ca^2+^ influx caused by the depolarizing stimulus with high extracellular K^+^. Frequency modulation-encoding oscillations are mediated by the process of CICR [65]. Because CSDCWs depended mainly on voltage-gated Ca^2+^ influx rather than the process of CICR, frequency modulation-encoding oscillations would not determine the transition from CASs to CSDCWs. It will be interesting to study the interactions between non-CSDCWs and CSDCWs, because that non-CSDCWs compromise a large number of synchronized astrocytes, which might affect the incidence of CSDCWs. However, we failed to elicit non-CSDCWs, which might be due to that in our model the internal Ca^2+^ dynamics does not implement the frequency modulation-encoding oscillations and that ATP diffusion in the ECS is not considered. Ca^2+^ in non-CSDCWs originates mainly from internal stores of astrocytes [18], [19], while Ca^2+^ in CSDCWs, according to our study, originates mainly from Ca^2+^ influx, and Ca^2+^ in internal stores also contributes to CSDCWs. Base on these findings, non-CSDCWs seem not to suppress the incidence of CSDCWs but to change their properties. This might be supported by the experiments that after depletion of the internal Ca^2+^ stores, CSDCWs could still be recorded but the amplitude of Ca^2+^ signal was reduced [21]. In contrast, CSDCWs might affect the occurrence of non-CSDCWs by taking up the process of CICR. Future experiments are needed to elucidate the interactions between non-CSDCWs and CSDCWs.

Due to changes in the membrane potential are difficult to model because of the many and complex processes involved, we and others [66] use a simplified model of using the K^+^ Nernst potential to approximate the membrane potential, but add a modulation factor. The value of the modulation factor was chosen based on the considerations: after adding the modulation factor, astrocyte membrane potential is within the range where CASs would occur [32]; according to experimental observations [43], [67], the frequency of CASs is significantly decreased when the temperature in this model increases from 20 to 37°C (Fig. S5). Ca^2+^ recording from astrocytes in vivo shows that astrocytes were either quiescent or responded with a few Ca^2+^ transients [12], [68], while Ca^2+^ transients occurred more frequently in slices prepared at 28°C [17]. When the slices were prepared at 37°C, no statistical difference was found in the percentage of active astrocytes and the frequency of Ca^2+^ events between the in vivo and in situ results [52]. Moreover, the frequency of CASs was showed to be temperature-dependent: from 20 to 37°C, CASs occurred frequently at low temperature and became less frequent at higher temperature [43], [67]. The mechanism underlying this would be the decreased activity of IP_3_R channels or the increased activity of SERCA at higher temperature [69], [70], which reduces the Ca^2+^ released from internal stores and the frequency of CASs. Another limitation is that we did not consider the volume of the extracellular space. Although the volume of astrocytes is not altered during CSD [71], the volume of the extracellular space is decreased. To model these Ca^2+^ signals more accurately, the changes of volume of the extracellular space need be taken into account.

This model could be improved at least in two aspects. First, Ca^2+^ waves in astrocytes are thought to be transmitted by gap junction or by extracellular diffusion of ATP [72], [73]. The former seems predominant in the neocortex [21], [74], and the latter in the archicortex and spinal cord [75], [76]. In the present model, the astrocytes are coupled by the transfer of IP_3_ through gap junction, and the ATP diffusion is not considered. Hence, the results in the present study are expected to be relevant to the brain structures in neocortex. To compare the properties of Ca^2+^ waves in different brain areas, the gap junction and ATP diffusion should be considered together. ATP-mediated Ca^2+^ waves have been modeled in the astrocyte network [31], [77]. However, because of the complex details in the ATP production and propagation, it’s not very easy to integrate these models into our model. Nevertheless, this part of the improvement will be the goal of our future work. Second, astrocytes communicate not only with themselves, but also with neurons, and Ca^2+^ signals in astrocytes could be affected by the neuronal activity [11], [16]. Transmitters released from neurons, for example, glutamate, should be considered to understand the Ca^2+^ signals, especially the CSDCWs [31].

In summary, we analyze the contributions of different Ca^2+^ flows to the generation of CASs and CSDCWs, and indicate different mechanisms underlying CSDCWs and non-CSDCWs. An experiment test could be done is to example the effects of Ca^2+^ influx, especially Ca^2+^ influx via VGCCs, on the generation and propagation of CSDCWs. Research on the different types of Ca^2+^ signals might help to understand the different ways by which astrocytes participate in the brain functions.

## Supporting Information

Figure S1 **The influence of CICR on CASs.** From (A) to (E), the inhibition of CICR is 0%, 30%, 70%, 90% and 100%, respectively. By inhibiting CICR gradually, the frequency and amplitude of CASs decrease, while the duration increases.
(TIF)

Figure S2 **The influence of SERCA on CASs.** From (A) to (E), the inhibition of SERCA is 0%, 20%, 50%, 70% and 90%, respectively. By inhibiting SERCA gradually, the amplitude and duration of CASs decrease, while the frequency increases. Ca^2+^ dynamics evolve into small oscillations before disappearing.
(TIF)

Figure S3 **The influence of VGCCs on CASs.** From (A) to (E), the inhibition of VGCCs is 0%, 20%, 30%, 40% and 50%, respectively. In the present of CASs, inhibiting VGCCs has little influence on the amplitude and duration of CASs, but great on frequency.
(TIF)

Figure S4 **Dynamics of CSD.** During CSD, K^+^ in the ECS (A) and in the ICS (B) is significantly increased and astrocytes are depolarized at successive astrocytes in the network (C). The bar illustrates the time of locally elevating *K*_o_ to evoke a CSD.
(TIF)

Figure S5 **The influence of temperature on the frequency of CASs.** From (A) to (D), the value of temperature used in the model is 20, 25, 31, and 36°C, respectively. As the temperature increases, the frequency of CASs decreases. (E) CASs occur frequently at low temperature and become less frequent at higher temperature.
(TIF)

Table S1 **The parameter values used in the model.**
(PDF)

Table S2 **The initial values of the variables.**
(PDF)

Materials S1 **Supporting materials that briefly describe the Ca^2+^ flows through the membrane of ER and the dynamics of IP_3_ in single astrocytes in the previous model of CASs.**
(DOC)
