# Supplementary figures and images for: Modeling the Contributions of Ca2+ Flows to Spontaneous Ca2+ Oscillations and Cortical Spreading Depression-Triggered Ca2+ Waves in Astrocyte Networks
Source: PLoS One. 2012 Oct 31;7(10):e48534. doi: 10.1371/journal.pone.0048534 (PMC3485305; doi:10.1371/journal.pone.0048534)

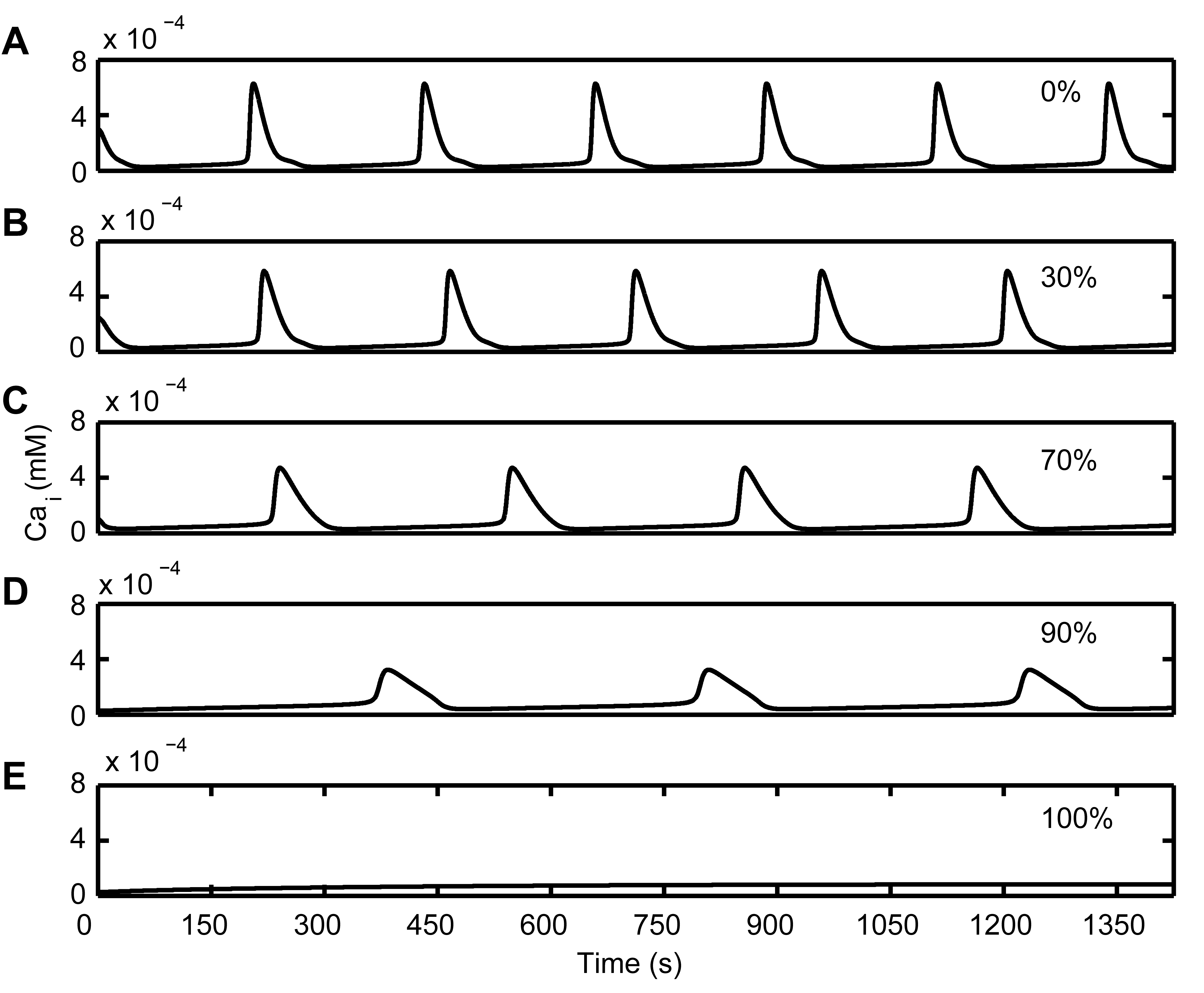

Supplement: Figure S1 — The influence of CICR on CASs. From (A) to (E), the inhibition of CICR is 0%, 30%, 70%, 90% and 100%, respectively. By inhibiting CICR gradually, the frequency and amplitude of CASs decrease, while the duration increases. (TIF) [file pone.0048534.s001.tif]

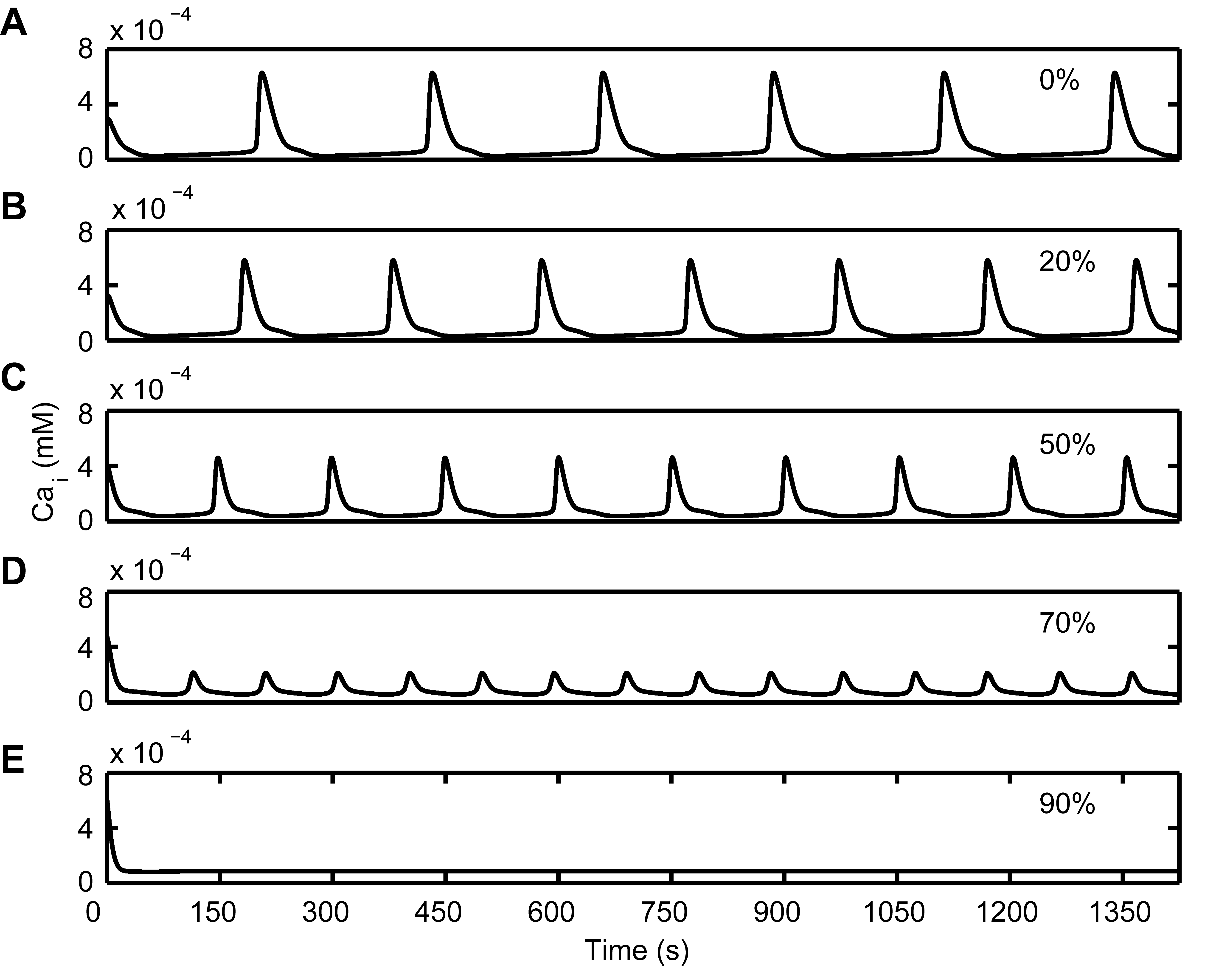

Supplement: Figure S2 — The influence of SERCA on CASs. From (A) to (E), the inhibition of SERCA is 0%, 20%, 50%, 70% and 90%, respectively. By inhibiting SERCA gradually, the amplitude and duration of CASs decrease, while the frequency increases. Ca2+ dynamics evolve into small oscillations before disappearing. (TIF) [file pone.0048534.s002.tif]

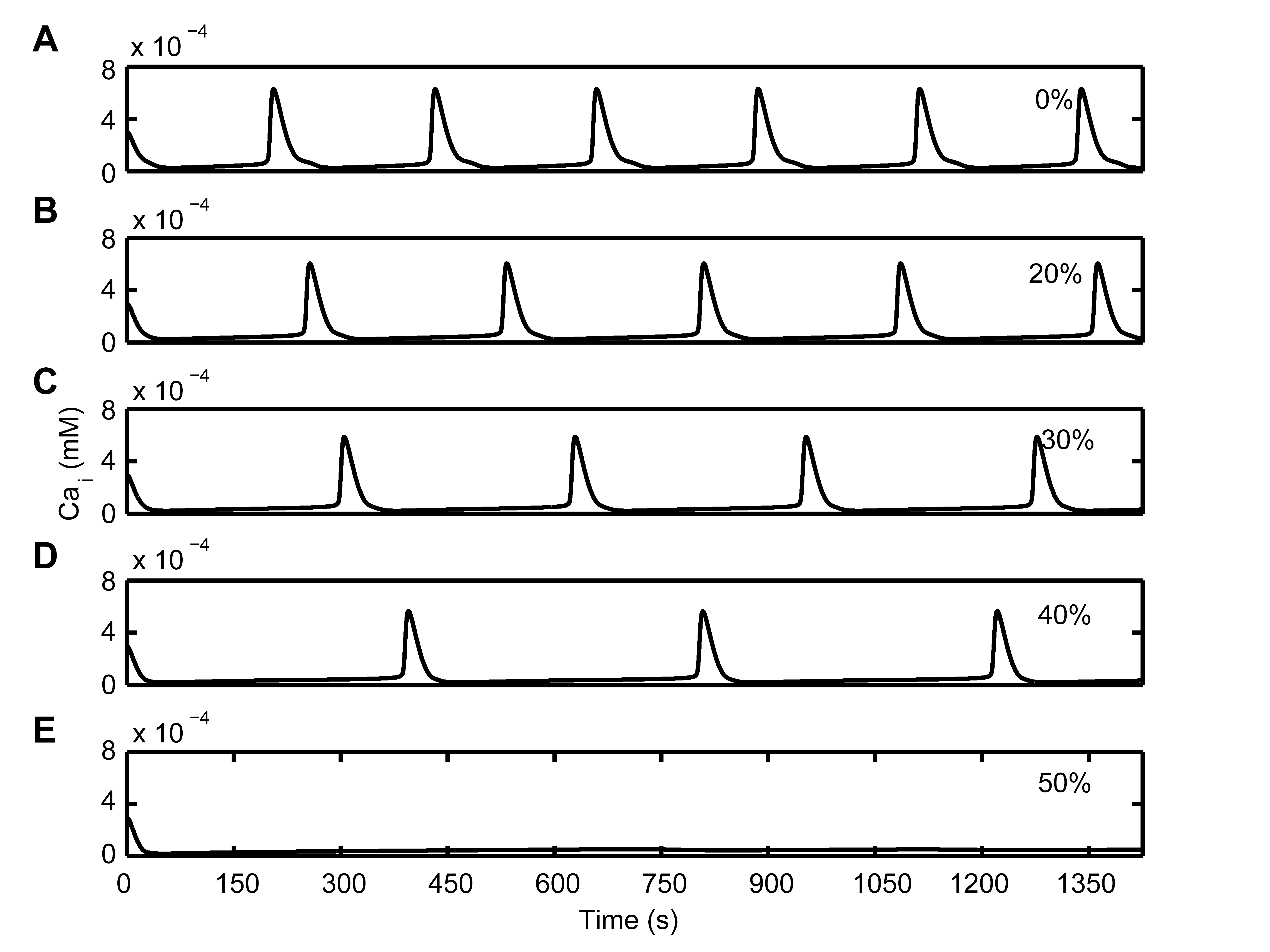

Supplement: Figure S3 — The influence of VGCCs on CASs. From (A) to (E), the inhibition of VGCCs is 0%, 20%, 30%, 40% and 50%, respectively. In the present of CASs, inhibiting VGCCs has little influence on the amplitude and duration of CASs, but great on frequency. (TIF) [file pone.0048534.s003.tif]

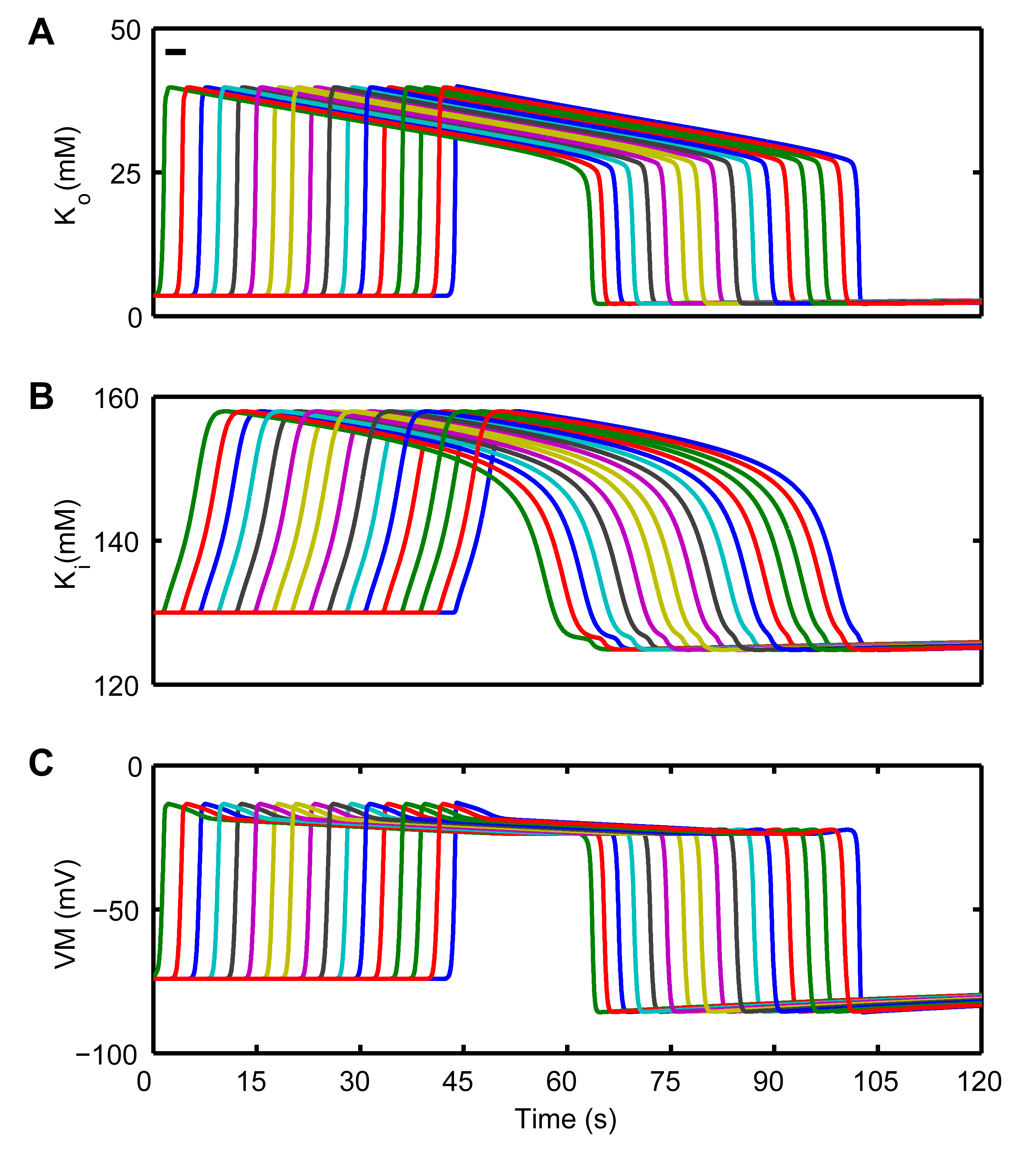

Supplement: Figure S4 — Dynamics of CSD. During CSD, K+ in the ECS (A) and in the ICS (B) is significantly increased and astrocytes are depolarized at successive astrocytes in the network (C). The bar illustrates the time of locally elevating K o to evoke a CSD. (TIF) [file pone.0048534.s004.tif]

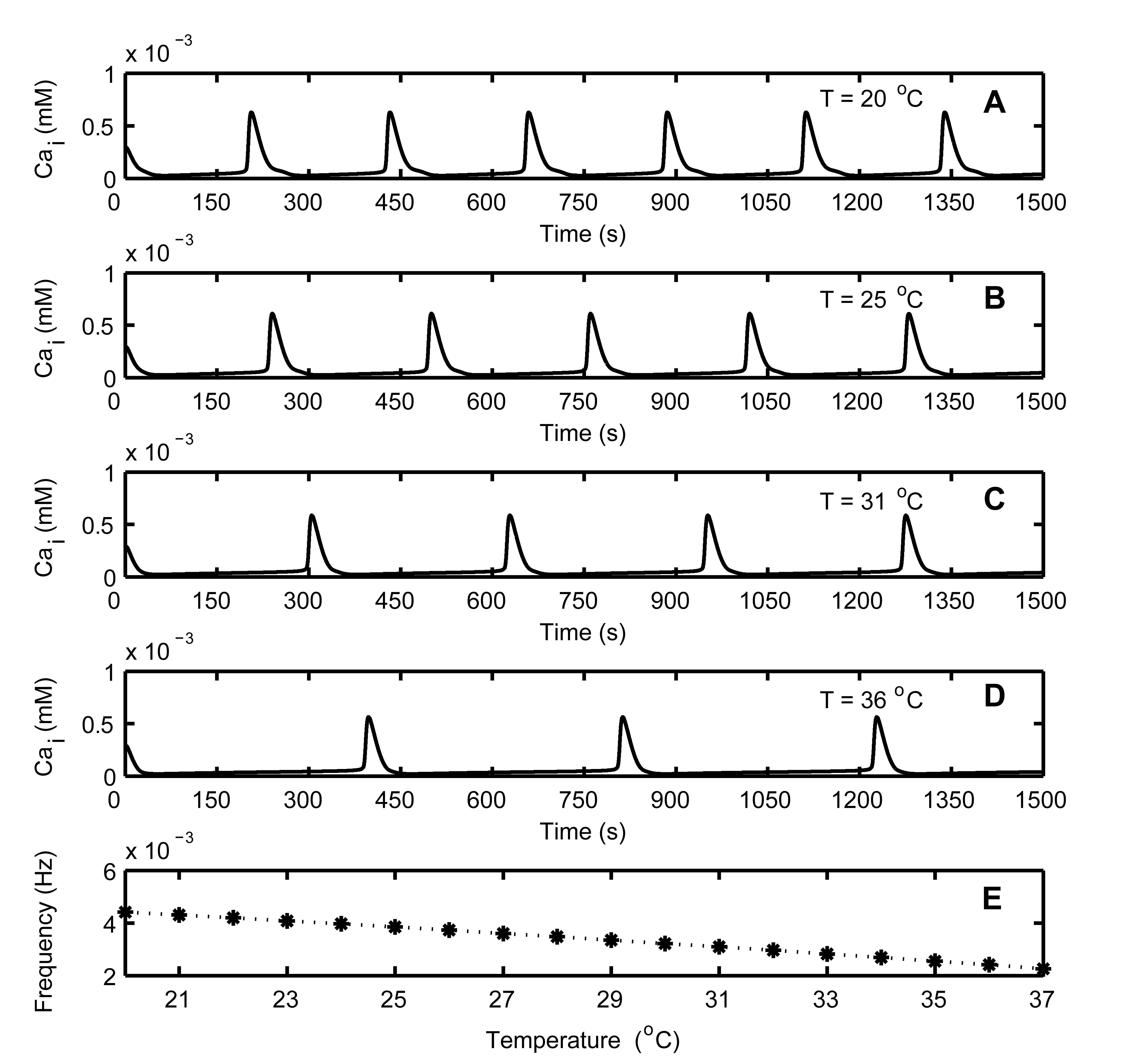

Supplement: Figure S5 — The influence of temperature on the frequency of CASs. From (A) to (D), the value of temperature used in the model is 20, 25, 31, and 36°C, respectively. As the temperature increases, the frequency of CASs decreases. (E) CASs occur frequently at low temperature and become less frequent at higher temperature. (TIF) [file pone.0048534.s005.tif]
