# Supplementary material for: Modeling the Contributions of Ca2+ Flows to Spontaneous Ca2+ Oscillations and Cortical Spreading Depression-Triggered Ca2+ Waves in Astrocyte Networks
Source: PLoS One. 2012 Oct 31;7(10):e48534. doi: 10.1371/journal.pone.0048534 (PMC3485305; doi:10.1371/journal.pone.0048534)
Supplement: Table S1 — The parameter values used in the model. (PDF) [file pone.0048534.s006.pdf]

Table S1. The parameter values used in the model.

Parameter	Definition	Value*
g_{VGCC}	Membrane conductance of the VGCCs	174.4 pS
V_{astro}	Volume of an astrocyte	5.233×10^{-13} l
g_{pump}	Membrane conductance of the pump	3 pS
L_{ext}	Rate of Ca^{2+} efflux from astrocytes	0.5 s^{-1}
M_{CICR}	Maximum flux rate of Ca^{2+} into the cytosol	40 s^{-1}
S_{act}	Activating affinity	1.5×10^{-4} mM
S_{inh}	Inhibiting affinity	1.5×10^{-4} mM
S_{IP3}	Half-saturation constant for IP_3 activation of IP_3R	1×10^{-4} mM
n	Hill coefficient	2.02
m	Hill coefficient	2.2
M_{SERCA}	Maximum flux across SERCA	0.015 mM/s
S_{SERCA}	Half-saturation constant for SERCA activation	0.0001 mM
L_{int}	Rate of Ca^{2+} efflux from the ER	0.5 s^{-1}
D_{Cai}	Diffusion coefficient for Ca^{2+} in cytosol	200 μm^2/s
D_{Cao}	Diffusion coefficient for Ca^{2+} in the ECS	790 μm^2/s
K_{omax}	Maximum K_o in ECS during CSD	40 mM
Ca_{orest}	Ca_o at the resting level	1 mM
M_{Ca}	Rate constant reflecting the influence of CSD	0.0063
S_{PLC}	Half-saturation constant for Ca^{2+} activation of PLC	3×10^{-4} mM
M_{deg}	Rate of IP_3 degradation	0.08 s^{-1}
M_{PLC}	Maximum production rate of PLC	5×10^{-5} mM/s
γ	Gap junction strength	2 s^{-1}
D_{IP3}	Diffusion coefficient for IP_3 in the ICS	280 μm^2/s
D_{Ko}	Diffusion coefficient for K^+ in the ECS	1960 μm^2/s
M_{KK}	Rate coefficient of K^+ dynamics in the ECS	-0.03 mM^{-2} s^{-1}
K_{orest}	K_o at the resting level	3.5 mM
K_θ	Threshold for triggering of CSD	10 mM
M_{KR}	Rate constant for recovery of K^+ in the ECS	2.56 mM^{-1} s^{-2}
M_R	Rate constant for decay of K^+ in the ECS	7.5 s^{-1}
M_{Ki}	Rate coefficient of K^+ dynamics in the ICS	-0.003 mM^{-2} s^{-1}
M_{io}	Rate constant for decay of K^+ in the ICS	0.2 s^{-1}
K_{irest}	K_i at the resting level	130 mM
$K_{i\theta}$	Threshold for fast K^+ elevation in the ICS	140 mM
K_{imax}	Maximum K_i in ICS during CSD	155 mM
A_{ki}	Rate constant for discharge of K^+ in the ICS	2.56 mM^{-1} s^{-2}
A_r	Rate constant for decay of K^+ in the ICS	0.75 s^{-1}
R	Ideal gas constant	8.31 J mol^{-1} K^{-1}
T	Absolute temperature	293 K
F	Faraday constant	96485 C/mol
z_K	Valence of K^+	1
z_{Ca}	Valence of Ca^{2+}	2
ε	Modulation factor	17 mV

*The values of parameters for the Ca2+ flows through the membrane of astrocytes or through the membrane of the ER were taken from the previous studies [1-4]. The values of parameters for the extracellular Ca2+ concentration and CSD were chosen in order to allow the generation of CSDCWs. With these parameters, the properties of CASs and CSDCWs in the simulations are consistent with the reported literature values [2,3,5,6].

References

1. Zeng S, Li B, Zeng S, Chen S (2009) Simulation of spontaneous Ca2+ oscillations in astrocytes mediated by voltage-gated calcium channels. Biophys J 97: 2429-2437.
2. Lavrentovich M, Hemkin S (2008) A mathematical model of spontaneous calcium(II) oscillations in astrocytes. J Theor Biol 251: 553-560.
3. Bennett MR, Farnell L, Gibson WG (2008) A quantitative model of cortical spreading depression due to purinergic and gap-junction transmission in astrocyte networks. Biophys J 95: 5648-5660.
4. Dronne MA, Boissel JP, Grenier E (2006) A mathematical model of ion movements in grey matter during a stroke. J Theor Biol 240: 599-615.
5. Tashiro A, Goldberg J, Yuste R (2002) Calcium oscillations in neocortical astrocytes under epileptiform conditions. J Neurobiol 50: 45-55.
6. Parri HR, Gould TM, Crunelli V (2001) Spontaneous astrocytic Ca2+ oscillations in situ drive NMDAR-mediated neuronal excitation. Nat Neurosci 4: 803-812.
