# Supplementary material for: Modeling the Contributions of Ca2+ Flows to Spontaneous Ca2+ Oscillations and Cortical Spreading Depression-Triggered Ca2+ Waves in Astrocyte Networks
Source: PLoS One. 2012 Oct 31;7(10):e48534. doi: 10.1371/journal.pone.0048534 (PMC3485305; doi:10.1371/journal.pone.0048534)
Supplement: Table S2 — The initial values of the variables. (PDF) [file pone.0048534.s007.pdf]

Table S2. The initial values of the variables.

Parameter	Description	Value (unit)
IP_3	IP_3 concentration in the ICS	0.0001 mM
Ca_i	Ca^{2+} concentration in the ICS	0.0001 mM
Ca_{ER}	Ca^{2+} concentration in the ER	0.00015 mM
Ca_o	Ca^{2+} concentration in the ECS	1 mM
K_o	K^+ concentration in the ECS	3.5 mM
K_i	K^+ concentration in the ICS	130 mM
P_i	Recovery for K^+ in the ICS	0 s^{-1}
R_k	Recovery for K^+ in the ECS	0 s^{-1}
