# Supplementary material for: Modeling the Contributions of Ca2+ Flows to Spontaneous Ca2+ Oscillations and Cortical Spreading Depression-Triggered Ca2+ Waves in Astrocyte Networks
Source: PLoS One. 2012 Oct 31;7(10):e48534. doi: 10.1371/journal.pone.0048534 (PMC3485305; doi:10.1371/journal.pone.0048534)
Supplement: Materials S1 — Supporting materials that briefly describe the Ca2+ flows through the membrane of ER and the dynamics of IP3 in single astrocytes in the previous model of CASs. (DOC) [file pone.0048534.s008.doc]

**Materials S1**

The supporting materials provide a brief description of our previous model [1], which simulated the voltage-gated calcium channels (VGCCs)-mediated spontaneous Ca^2+^ oscillations (CASs). Three kinds of Ca^2+^ flows through the membrance of endoplasmic reticulum (ER) in astrocytes, including Ca^2+^ released from calcium-induced calcium release (CICR), Ca^2+^ uptaken into the ER and leak flux through the membrance of ER, were considered in this model.

The Ca^2+^ flow which was released from CICR was determined by the equation:

(S1)

where, *M*_CICR_, *S*_act_, *S*_inh_, and *S*_IP3_ are constants, and *n* and *m* are Hill coefficients. *IP*_3_ represents the IP_3_ concentration in the intracellular space (ICS). *Ca*_i_ and *Ca*_ER_ represent the Ca^2+^ concentration in the ICS and in the ER, respectively.

The Ca^2+^ flow which was uptaken into the ER via the sarcoplasmic/endoplasmic reticulum Ca^2+^ ATPase (SERCA) was given by:

(S2)

where, *M*_SERCA_ and *S*_SERCA_ are constants.

Leak flux into the ICS was considered using the following equation:

(S3)

where, *L*_int_ is the rate of Ca^2+^ efflux from the ER to the astrocytic cytosol.

IP_3_ in the ICS was used to activate the process of CICR and was catalyzed by phospholipase C (PLC):

(S4)

where, *M*_PLC_, *M*_deg_ and *S*_PLC_ are constants. The first term on the right of Eq. (S4) represents the production of IP_3_ (*IP*_pro_) and the second term represents its degradation (*IP*_deg_).

The parameter values used in the model are shown in Table S1 in the Supporting Information.

**References**

1. Zeng S, Li B, Zeng S, Chen S (2009) Simulation of spontaneous Ca2+ oscillations in astrocytes mediated by voltage-gated calcium channels. Biophys J 97: 2429-2437.
